# Efficacy and Safety of Teriparatide in Improving Fracture Healing and Callus Formation: A Systematic Review

**DOI:** 10.7759/cureus.37478

**Published:** 2023-04-12

**Authors:** Chaitanya S Puvvada, Faiza H Soomro, Hafsa A Osman, Merna Haridi, Natalie A Gonzalez, Sana M Dayo, Umaima Fatima, Aaiyat Sheikh, Sai Sri Penumetcha

**Affiliations:** 1 Internal Medicine, California Institute of Behavioral Neurosciences & Psychology, Fairfield, USA; 2 Internal Medicine, Gayatri Vidya Parishad Institute of Health Care and Medical Technology, Visakhapatnam, IND; 3 General Surgery, California Institute of Behavioral Neurosciences & Psychology, Fairfield, USA; 4 General Surgery, NineWells Hospital, NHS Tayside, Dundee, GBR; 5 Pediatrics, California Institute of Behavioral Neurosciences & Psychology, Fairfield, USA; 6 Medical Education, Saint Martinus University, Curacao, CUW; 7 Obstetrics and Gynecology, California Institute of Behavioral Neurosciences & Psychology, Fairfield, USA; 8 Internal Medicine, Era's Lucknow Medical College & Hospital, Lucknow, IND; 9 General Medicine, California Institute of Behavioral Neurosciences & Psychology, Fairfield, USA; 10 General Medicine, Chalmeda Anand Rao Institute of Medical Sciences, Karimnagar, IND

**Keywords:** nonunion, delayed union, callus, fracture, parathyroid hormone, teriparatide

## Abstract

Fracture nonunion remains a great challenge for orthopedic surgeons. Some bone fractures don't heal promptly, resulting in delayed unions and nonunions, and there is a need for an additional surgical procedure. Previous research has shown that teriparatide, a type of synthetic parathyroid hormone, can promote the formation of callus and lead to healing in individuals with delayed or non-healing bone fractures. Limited systematic reviews exist that examine the use of teriparatide in cases of delayed healing or non-healing bone fractures, which have their limitations. In this review, we overcome those limitations by including prospective studies, retrospective studies, case reports, and case series together.

A systematic search of the literature was conducted in both PubMed and Google Scholar up to September of the year 2022. The studies included in our research included adult patients (over the age of 16) diagnosed with delayed union or nonunion of any bone in the body (flat bone, long bone, short bone, or irregular bone). The studies were limited to those written in English. The outcomes that were tracked and recorded include the healing of the fracture and any negative side effects or adverse events.

The initial search yielded 504 abstracts and titles. After reviewing these, 32 articles were selected for further analysis, which included 19 case reports, five case series, two retrospective studies, and six prospective studies. Studies included daily (20 micrograms) or weekly (56.5 micrograms) subcutaneous administration of teriparatide. The duration of follow-up for these studies varied from three to 24 months.

Based on the available research, it appears that administering teriparatide subcutaneously is a safe treatment option for delayed healing and non-healing bone fractures, with very few to no reported negative side effects. Using teriparatide for induction of callus formation and treating delayed and nonunions is highly safe and effective.

## Introduction and background

About five to ten percent of bone fractures do not heal as they are expected to in the time interval they are normally supposed to heal [1,2]. This can lead to delayed healing or non-healing bone fractures, which require additional hospital treatment and can cause significant physical, mental, and financial difficulties for patients. Despite advances in treatment methods, many of the current options are surgical in nature and involve reopening the fracture site and risking post-operative infections, prolonging hospital stay, and other surgical complications. Therefore, there is a need for a more conservative treatment modality that can stimulate fracture unions with minimal intervention and minimal adverse effects.

Parathyroid glands located in the posterior region of the thyroid gland secrete the parathyroid hormone, which is also called parathormone. Parathormone is one of the key regulators of calcium homeostasis and the metabolism of calcium in the body. Although the parathyroid hormone is thought to stimulate the function of osteoclasts ("bone-eating cells"), causing bone resorption and bone loss, bone loss is observed only to be in hyperparathyroidism, which results in higher levels of circulating parathormone.

Intermittent administration of the parathyroid hormone (PTH) has been shown to increase bone mass due to its anabolic effects dominating the catabolic effects [3]. Teriparatide, a synthetic PTH analog, which contains the first 34 residues of parathyroid hormone (PTH 1-34), is often used for treating osteoporosis. Teriparatide is considered to be the most potent of osteoporosis therapies due to its marked anabolic effects [4]. The existing basic science data suggest teriparatide accelerates chondrocyte recruitment and differentiation, which are essential processes in early enchondral ossification [5,6].

Numerous clinical studies have reported the efficacy of teriparatide in promoting fracture healing. Teriparatide works by stimulating osteoblasts and reducing osteoblast apoptosis, resulting in an increased osteoblast life span [7]. Teriparatide also works by increasing callus formation and improving the mechanical strength of bone at the fracture site [8]. Most reviews in this area focus on osteoporosis; the present review is the most recent and systematic on teriparatide and its effect on bone healing [9].

## Review

Methods

This systematic review was conducted in accordance with the Preferred Reporting Items for Systematic Review and Meta-Analysis (PRISMA) guidelines [10].

Participants

The studies that we analyzed in our research included adult patients (over the age of 16) diagnosed with delayed union or nonunion of any bone in the body (long bone, flat bone, short bone, or irregular bone) or treatment modality used for initial fracture treatment (surgical or conservative).

Interventions

The interventions studied included the administration of teriparatide via any route of administration (but generally by subcutaneous route) at any dose and frequency.

Outcomes

The outcomes measured and recorded were the healing of the fracture and any negative side effects or adverse events.

Study Characteristics

All types of study designs, such as case reports, case series, and retrospective and prospective studies, were included in the research.

Information sources

Our study utilized a systematic literature search with PubMed and Google Scholar to gather relevant articles. The first search was performed on September 10, 2022. The second search was conducted on September 25, 2022.

Search strategy

A combination of the following free words and Medical Subject Headings (MeSH) terms were used: "teriparatide", "parathyroid hormone", "PTH analogs", "delayed union", "nonunion", and "fracture healing". Boolean operators, including "AND" and "OR" were used. A systematic search of the literature was conducted in both PubMed and Google Scholar up to September 2022. Table 1 shows the databases used and the search strategy used for each database.

**Table 1:** Databases and search strategy PTH - parathyroid hormone

Database	Search strategy
PubMed	(teriparatide OR parathyroid hormone OR PTH analogs) AND (fracture healing OR nonunion OR delayed union)
Google Scholar	(teriparatide OR parathyroid hormone OR PTH analogs) AND (fracture healing OR nonunion OR nonunion OR delayed union)

Quality check

The quality of the included studies was appraised using the following tools, shown in Table 2.

**Table 2:** Quality appraisal tools used RCT - randomized controlled trial, JB - Joanna Briggs, PRISMA - Preferred Reporting Items for Systematic Reviews and Meta-Analyses, SANRA - Scale for the Assessment of Narrative Review Articles

Type of study	Quality appraisal tool used
Randomized controlled trials	Cochrane bias assessment tool
Non-RCT and observational studies	Newcastle Ottawa tool
Case reports	JB check tool
Systematic reviews	PRISMA checklist
Research paper without clear methods section	SANRA checklist

Results

Literature Search

**Figure 1:**
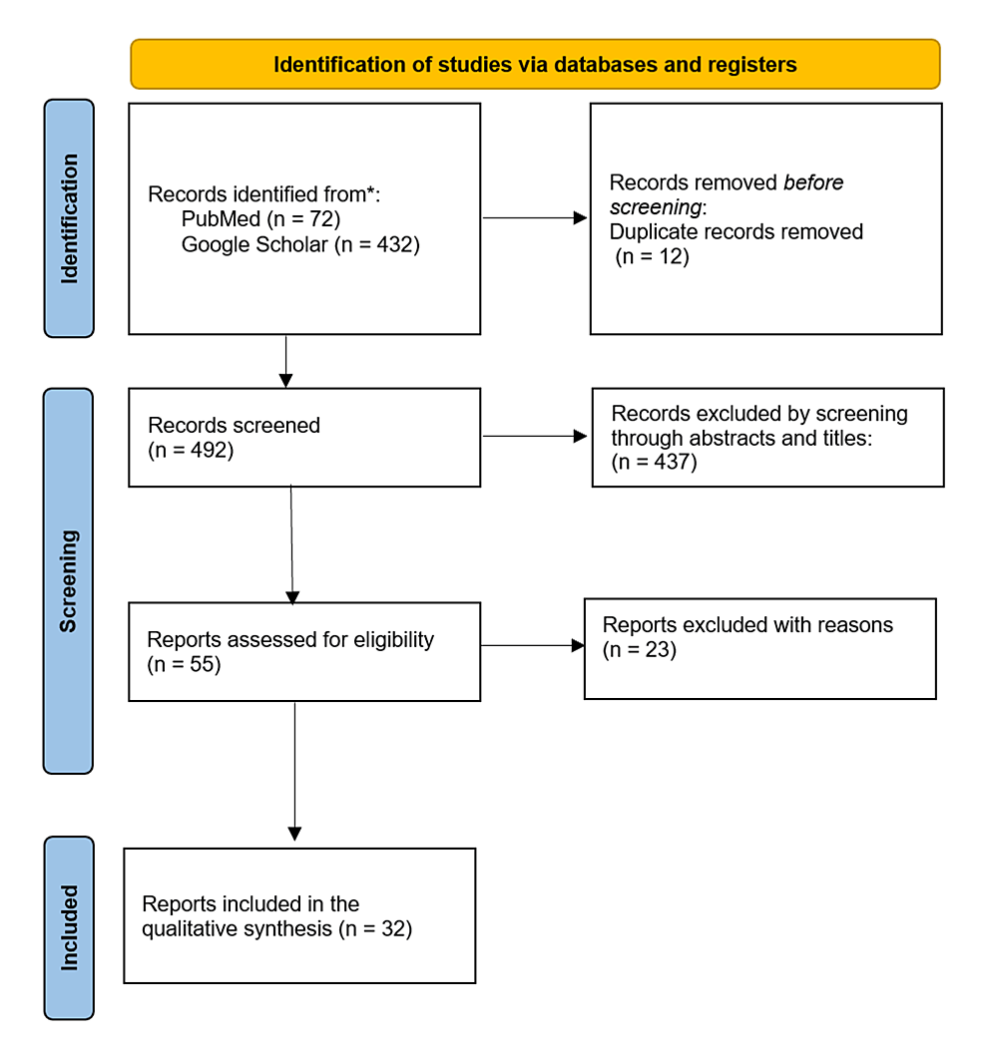
PRISMA flowchart of selected articles on teriparatide and bone healing PRISMA - Preferred Reporting Items for Systematic Reviews and Meta-Analyses

A total of 504 publications were retrieved based on our search criteria. Of these, 472 were excluded during the screening process, and 32 articles were finally included. To further illustrate the selected articles used, the PRISMA flowchart shows the process in more detail (Figure 1)[10].

Study Characteristics

Out of the 32 articles reviewed, we identified 19 case reports, five case series, two retrospective studies, and six prospective studies. The number of participants in each study ranged from one to 159. In total, 572 participants were included in this review. The participants included both males and females. Table 3 shows the study characteristics of this systematic review.

**Table 3:** Study characteristics PFN - proximal femoral nail, USA - United States of America

Author(s)	Year of publication	Country of study	Study design	Number of subjects	Male:female	Age (in years)
Almirol et al., 2016 [11]	2016	United Kingdom	Prospective study	14 (6 in treatment group, 8 in placebo group)	0:14	21-45
Aspenberg et al., 2010 [12]	2010	Sweden	Prospective study	27	0:27	>50
Baillieul et al., 2016 [13]	2016	France	Case report	1	0:1	36
Bhandari et al., 2016 [14]	2016	USA	Prospective study	159 (78 teriparatide, 81 placebo)	Not specified (both male and female)	>50yrs
Bednar et al., 2016 [15]	2016	Canada	Case report	1	0:01	70
Coppola et al., 2015 [2]	2015	Italy	Case series	4	4:0	31.75 (mean)
Chintamaneni et al., 2010 [16]	2010	USA	Case report	1	1:0	67
Fukuda et al., 2014 [17]	2014	Japan	Case report	1	0:1	74
Gende et al., 2020 [18]	2020	USA	Case report	1	0:1	Not specified
Gianotti et al., 2013 [19]	2013	Italy	Case report	1	0:1	80
Huang et al., 2015 [20]	2015	Korea	Retrospective study	81 (31 received teriparatide, 50 did not receive teriparatide)	28:53	65 to 92 (mean 82.3)
Ismailidis et al., 2021 [21]	2021	Switzerland	Case series	5	2:3	50-77 years (mean 61.8)
Kastirr et al., 2018 [22]	2018	Germany	Case series	32	15:17	22-83
Kastirr et al., 2016 [23]	2016	Germany	Case report	1	1:0	49
Kim et al., 2018 [24]	2018	Korea	Prospective study	96 (50 only proximal femoral nail; 46 PFN and teriparatide)	44:52	65-99 (avg 82yrs)
Kim et al., 2019 [25]	2019	Korea	Retrospective study	112 (60 PFN alone; 52 PFN and teriparatide)	Not specified	>65yrs
Lee et al., 2012 [26]	2012	South Korea	Case series	3	2:1	29-64 (mean 43.67)
Mancilla et al., 2015 [27]	2015	USA	Case series	6	1:5	19-64
Matsumoto et al., 2014 [28]	2014	Japan	Case report	1	1:0	70
Mitani et al., 2013 [29]	2013	Japan	Case report	1	0:1	88
Nozaka et al., 2014 [30]	2014	Japan	Case report	1	1:0	56
Ochi et al., 2013 [31]	2013	Japan	Case report	1	0:1	74
Oteo-Alvaro et al., 2010 [32]	2010	Spain	Case report	1	1:0	32
Pola et al., 2017 [33]	2017	Italy	Case report	1	0:1	73
Raghavan et al., 2012 [34]	2012	India	Case series	2	0:2	35, 40
Rubery et al., 2010 [35]	2010	USA	Case series	3	0:3	85.67 (mean)
Saraf et al., 2017 [36]	2017	India	Prospective study	20	10:1	>25
Tachiiri et al., 2014 [37]	2014	Japan	Case report	2	0:2	72
Tamai et al., 2013 [38]	2013	Japan	Case report	1	0:1	25
Tsai et al., 2019 [39]	2019	Taiwan	Case report	1	0:1	60
Wu et al., 2012 [40]	2012	China	Case report	1	0:1	73
Xiaofeng et al., 2017 [41]	2017	China	Case report	1	1:0	44
Yu et al., 2017 [1]	2017	China	Case report	1	1:0	45

Interventions

Table 4 shows the interventions performed in each article.

**Table 4:** Interventions IU - international units, mg - milligrams, LIPUS - low-intensity pulsed ultrasound, PFN - proximal femoral nail

Study	Interventions	Dose of teriparatide in micrograms	Given daily/weekly	Route of administration of teriparatide	Duration of treatment
Almirol et al., 2016 [11]	Teriparatide or placebo	20	Daily	Subcutaneous injection	8 weeks
Aspenberg et al., 2010 [12]	Teriparatide	20 or 40	Daily	Subcutaneous injection	8 weeks
Baillieul et al., 2016 [13]	Teriparatide	20	Daily	Subcutaneous injection	6 months
Bhandari et al., 2016 [14]	Teriparatide or placebo plus supplemented calcium (<1000mg/day) and vitamin D (<4000IU/day)	20	Daily	Subcutaneous injection	6 months
Bednar et al., 2016 [15]	Teriparatide	20	Daily	Subcutaneous injection	3 months
Coppola et al., 2015 [2]	Teriparatide	20	Daily	Subcutaneous injection	5 (3-9) months
Chintamaneni et al., 2010 [16]	Teriparatide	20	Daily	Subcutaneous injection	9 months
Fukuda et al., 2014 [17]	Teriparatide	56.5	Weekly	Subcutaneous injection	6 months
Gende et al., 2020 [18]	Teriparatide	20	Daily	Subcutaneous injection	12 weeks
Gianotti et al., 2013 [19]	Teriparatide	20	Daily	Subcutaneous injection	3 months
Huang et al., 2015 [20]	Teriparatide and no teriparatide	20	Daily	Subcutaneous injection	18 months
Ismailidis et al., 2021 [21]	Teriparatide (calcium and vitamin D supplemented in insufficient cases)	20	Daily	Subcutaneous injection	a) 24 months, b) 18 months, c) 12 months, d) 21 months, e) 9 months
Kastirr et al., 2018 [22]	Teriparatide	20	Daily	Subcutaneous injection	4-10 weeks
Kastirr et al., 2016 [23]	Teriparatide	20	Daily	Subcutaneous injection	4 months
Kim et al., 2018 [24]	PFN plus teriparatide to treatment group and only PFN for control group	56.5	Weekly	Subcutaneous injection	8 weeks
Kim et al., 2019 [25]	Teriparatide after PFN fixation	20	Daily	Subcutaneous injection	8 weeks
Lee et al., 2012 [26]	Teriparatide	20	Daily	Subcutaneous injection	3-9 months
Mancilla et al., [27]	Teriparatide	20	Daily	Subcutaneous injection	3-9 months
Matsumoto et al., 2014 [28]	Teriparatide	20	Daily	Subcutaneous injection	6months
Mitani et al., 2013 [29]	Teriparatide	56.5	Weekly	Subcutaneous injection	9 months
Nozaka et al., 2014 [30]	Teriparatide + LIPUS	Not specified	Not specified	Not specified	6 months
Ochi et al., 2013 [31]	Teriparatide	20	20	Subcutaneous injection	6 months
Oteo-Alvaro et al., 2010 [32]	Teriparatide	20	Daily	Subcutaneous injection	5 months
Pola et al., 2017 [33]	Teriparatide	20	Daily	Subcutaneous injection	3 months
Raghavan et al., 2012 [34]	Teriparatide, vitamin D3 50,000 IU, calcium citrate 2000mg	20	Daily	Subcutaneous injection	4 weeks
Rubery et al., 2010 [35]	Teriparatide	20	Daily	Subcutaneous injection	Not specified
Saraf et al., 2017 [36]	Teriparatide	20	Daily	Subcutaneous injection	10-16 weeks
Tachiiri et al., 2014 [37]	Teriparatide	56.5	Weekly	Subcutaneous injection	4 months
Tamai et al., 2013 [38]	Teriparatide and alfacalcidiol	20	Daily	Subcutaneous injection	Not specified
Tsai et al., 2019 [39]	Teriparatide	20	Daily	Subcutaneous injection	6 months
Wu et al., 2012 [40]	Teriparatide, calcium 1000mg/day, vitamin D 400IU/day	20	Daily	Subcutaneous injection	12 weeks
Xiaofeng et al., 2017 [41]	Teriparatide	20	Daily	Subcutaneous injection	8 months
Yu et al., 2017 [1]	Teriparatide	20	Daily	Subcutaneous injection	9 months

Diagnosis

Table 5 shows the diagnosis of patients included in the respective articles.

**Table 5:** Diagnosis

Author(s)	Diagnosis
Almirol et al., 2016 [11]	Lower extremity stress fractures
Aspenberg et al., 2010 [12]	Dorsally dislocated distal radial fracture
Baillieul et al., 2016 [13]	Bilateral sacral stress fracture complicated by delayed union
Bhandari et al., 2016 [14]	Femoral neck fracture
Bednar et al., 2016 [15]	Type 3 odontoid process fracture nonunion
Coppola et al., 2015 [2]	Lower limb nonunion
Chintamaneni et al., 2010 [16]	Sternal nonunion
Fukuda et al., 2014 [17]	Delayed union of atypical subtrochanteric fracture
Gende et al., 2020 [18]	Delayed union of ischioacetabular stress fracture
Gianotti et al., 2013 [19]	Distal femur metaphysis fracture - atrophic nonunion
Huang et al., 2015 [20]	Unstable per trochanteric fractures
Ismailidis et al., 2021 [21]	a) atypical femoral fracture, b) left clavicle fracture, c) periprosthetic humeral fracture, d) open femoral fracture + closed tibial and fibular fractures, e) bilateral open tibial and fibular fractures
Kastirr et al., 2018 [22]	Pilon tibial fracture nonunion (n = 16), distal crurale fracture nonunion (n = 2), femoral fracture nonunion (n = 8), metatarsal fracture nonunion (n = 1), distal humerus fracture nonunion (n = 1), olecranon fracture nonunion (n = 1), distal radius fracture nonunion (n = 1)
Kastirr et al., 2016 [23]	Aseptic delayed union of a distal lower leg fracture
Kim et al., 2018 [24]	Unstable intertrochanteric femoral fractures and osteoporosis
Kim et al., 2019 [25]	Unstable intertrochanteric femoral fractures and osteoporosis
Lee et al., 2012 [26]	Femur neck and shaft fractures - oligotrophic/ atrophic nonunions
Mancilla et al., [27]	Femoral and tibial shaft - atrophic nonunion
Matsumoto et al., 2014 [28]	Delayed union of lumbar vertebral fracture with diffuse idiopathic skeletal hyperostosis
Mitani et al., 2013 [29]	Femur neck fracture - atrophic nonunion
Nozaka et al., 2014 [30]	Femoral fracture shaft nonunion
Ochi et al., 2013 [31]	Periprosthetic fracture - atrophic nonunion
Oteo-Alvaro et al., 2010 [32]	Atrophic humeral shaft non union
Pola et al., 2017 [33]	Type 2 dens nonunion fractures
Raghavan et al., 2012 [34]	Metatarsal stress fractures
Rubery et al., 2010 [35]	Type 3 odontoid fractures non union
Saraf et al., 2017 [36]	Delayed unions, periprosthetic and osteoporotic fractures.
Tachiiri et al., 2014 [37]	Delayed union of fracture of right foot
Tamai et al., 2013 [38]	Nonunion of ankle fusion site, type 1 diabetes, severe osteoporosis, femoral shaft fracture
Tsai et al., 2019 [39]	Femur shaft fracture - atrophic nonunion
Wu et al., 2012 [40]	Sacral and pubic insufficiency fractures
Xiaofeng et al., 2017 [41]	Tibial and Femoral fracture - hypertrophic nonunion
Yu et al., 2017 [1]	Femoral shaft fracture - hypertrophic nonunion

Results

Table 6 shows the results of each article used in this systematic review.

**Table 6:** Results TPTD - teriparatide, mcg - micrograms, CT - computed tomography scan, SD - standard deviation, µg - micrograms

Author(s)	Mean time between initial fracture and teriparatide (in months)	Treatment results	Mean time for union	Side effects	Follow-up
Almirol et al., 2016 [11]	1	The TPTD-treated group showed a greater tibia cortical area and thickness compared to the placebo-treated group as early as eight weeks of treatment.	Not specified	None	2 months
Aspenberg et al., 2010 [12]	5.8 ± 2.3	The study found that a dose of 20 µg of teriparatide was effective in reducing the time to healing in three out of four cortices, compared to taking a placebo. However, a higher dose of 40 µg did not show the same positive results.	7.2 and 8.6 weeks for 20mcg and 40mcg groups respectively	Mild nausea in a single patient.	11 months
Baillieul et al., 2016 [13]	24	Patient was asymptomatic and on CT, re-mineralization was described.	6 months	None	6 months
Bhandari et al., 2016 [14]	167 days	Teriparatide did not decrease the risk of revision surgery or improve radio-graphic signs of fracture healing compared with the placebo.	84% of teriparatide group showed union at 12 months	None	12 months
Bednar et al., 2016 [15]	3	Complete fracture-site healing after six months (three months after discontinuing teriparatide therapy)	6 months	None	6 months
Coppola et al., 2015 [2]	9.5	One individual had a full bone healing within three years. Out of three other individuals, they returned to their normal activities between eight to 12 months, with an average of 10 months.	10 months	None	8 months to 5 years
Chintamaneni et al., 2010 [16]	Not reported	The fractures fully healed within nine months.	9 months	None	6 months
Fukuda et al., 2014 [17]	5	Complete union in three months	3 months	None	6 months
Gende et al., 2020 [18]	Not specified	Complete union in three months	3 months	None	3 months
Gianotti et al., 2013 [19]	7	Complete union in three months.	3 months	None	3 months
Huang et al., 2015 [20]	<1	The average time for the fractures to heal was longer in hips that did not receive teriparatide treatment (14.3 ± 2.8 weeks) compared to those that did (11.2 ± 1.6 weeks).	11.2 ± 1.6 weeks in teriparatide-treated group	Less femoral shortening and less varus collapse when compared with placebo group	18 months
Ismailidis et al., 2021 [21]	10.4	a) gradual callus build-up as well as pain reduction was noted in further follow-ups.	14.4	None	14.4 months
		b) clinical and radiological bone union was achieved six months postoperatively			
		c) clinical and radiological bone union was achieved 12 months postoperatively			
		d) the fracture gap showed gradual callus build-up, a radiological and clinical union was achieved.			
		e) clinical healing and radiological signs of bone union were achieved in 18 months.			
Kastirr et al., 2018 [22]	24.3 ± 17.8	All 30 patients achieved successful bone healing and regained full weight-bearing capacity, with an average treatment time of 4.1 ± 1.5 months. However, two patients did not show improvement despite treatment for 8 weeks.	4.1 ± 1.5(2 to 6 months)	None	4.1 +- 1.5 (2 - 6)
Kastirr et al., 2016 [23]	7	Bone growth across the fracture gap was observed four months after therapy was completed.	6 months	None	6 months
Kim et al., 2018 [24]	<1	There was no difference between two groups with respect to radiographic fracture healing at the final follow-up.	12.3 ± 6.4 weeks	None	19 months
Kim et al., 2019 [25]	Not specified	The mean time to fracture healing post-operatively was 14.8 weeks (SD 7.1) and 12.1 weeks (SD 6.4) in placebo group and teriparatide group, respectively.	12.1 ± 6.4 weeks	The frequency of patients reporting postoperative complications was also markedly reduced in the teriparatide-treated groups	6 months
Lee et al., 2012 [26]	20	The fractures fully healed between six to twelve months, with an average of 8.7 months, after treatment was discontinued.	13.7 months	None	9 – 15 months
Mancilla et al., [27]	13	Complete union in three to nine months in five out of six patients.	6.4 months in 5 subjects	None	3 – 9 months
Matsumoto et al., 2014 [28]	Not reported	Complete union in two months.	2 months	None	6 months
Mitani et al., 2013 [29]	13	Complete union in five months.	5 months	None	5 months
Nozaka et al., 2014 [30]	6	Patients were able to bear weight and had complete bone healing after six months.	6 months	None	6 months
Ochi et al., 2013 [31]	9	New bone filling between the fracture gap after five months.	6 months	None	6 months
Oteo-Alvaro et al., 2010 [32]	6	Fracture healing was achieved in five months.	5 months	None	6 months
Pola et al., 2017 [33]	6	A CT scan taken three months after treatment with Teriparatide showed complete healing of the fracture.	3 months	None	3 months
Raghavan et al., 2012 [34]	Case 1: six weeks	Case 1: four weeks into therapy with teriparatide, repeat imaging revealed evidence of bony callus and new bone formation.	1 month	None	1 month
	Case 2: ten weeks	Case 2: at the end of four weeks, repeat imaging revealed a fracture that was well healed with callus formation seen			
Rubery et al., 2010 [35]	4.7	Two months after discontinuation, four months after beginning teriparatide	2.5 months	None	4 months
Saraf et al., 2017 [36]	Teriparatide started immediately in periprosthetic fractures and in delayed union once the diagnosis was established	The treatment of delayed union fractures with teriparatide was associated with an average time of 12 weeks for bone growth across the fracture gap. For periprosthetic fractures, the average healing time was 12.6 weeks.	12.6 weeks	None	6 months
Tachiiri et al., 2014 [37]	4	Complete union in four months.	4 months	None	4 months
Tamai et al., 2013 [38]	3	Complete healing of femoral shaft fracture in 12 weeks, complete healing of ankle in 12 weeks	3 months	None	12 weeks
Tsai et al., 2019 [39]	6 months	After three months, X-ray images showed the presence of bone bridges and a decreased fracture gap between fragments.	11 months (teriparatide discontinued after five months)	None	12 months
Wu et al., 2012 [40]	Immediately after diagnosis	At three months' follow-up, the pain had subsided completely, with abundant callus formation on rami fracture. The fractures showed good consolidation at the end of 18 months	18 months	None	18 months
Xiaofeng et al., 2017 [41]	11	Complete fracture union after 12 months	12 months	None	12 months
Yu et al., 2017 [1]	25	Complete union after 15 months of the discontinuation of	24 months	None	24 months

Discussion

Currently, teriparatide has been widely proven to be effective in treating osteoporosis. However, there is an ongoing debate over whether teriparatide can improve fracture healing. This is not the first systematic review to examine the effect of teriparatide on fracture healing [9]. Fracture nonunion and delayed unions are devastating complications resulting from impaired bone healing. These conditions are characterized by pain and functional limitations, often resulting in decreased quality of life. Patients often respond differently to treatment for nonunions, making this condition very difficult to treat. A comparison between the results of all the considered studies has been described in Table 6.

In addition to six prospective studies and two retrospective investigations, this evaluation includes 32 research, primarily case reports and case series. In conclusion, teriparatide was found to be effective in treating nonunion without any negative side effects during the follow-up period, indicating that using it to treat nonunion is safe. However, the specific ways in which it promotes healing in patients with delayed union and nonunion have yet to be fully understood. Therefore, future research that examines the molecular processes underlying teriparatide's anabolic actions is necessary. From the case reports considered in this review, we found that the subjects with delayed and nonunions had achieved clinical union. Most of the interventions in these case studies included daily administration of 20 mcg of teriparatide, which suggests that teriparatide, when administered continuously, may also have anabolic effects on improving fracture healing.

A case series by Coppola et al. reported that teriparatide was effective in treating four cases of nonunions after open reduction and internal fixation of lower limb fractures. The patients had sufficient bone growth at the site of the nonunion, and they achieved both clinical and radiographic union. This study adds to the growing body of evidence that suggests teriparatide may be effective in the treatment of nonunions of lower limb fractures [2].

Similarly, a case study by Yu et al. treated a 45-year-old male with a nonunion of a femoral fracture using teriparatide for nine months. The patient received a daily dose of 20 mcg of teriparatide, and the treatment resulted in a fracture union. Additionally, there were no reported side effects. This case adds to the evidence that teriparatide may be effective in treating nonunion of femoral fractures with a good safety profile [3].

Prospective Studies

A total of six prospective studies were included in this review article, and five out of six showed a positive result. A prospective study done by Almirol et al. showed a positive result. The teriparatide-treated group showed a greater tibia cortical area and thickness compared to the placebo-treated group as early as eight weeks of treatment [11]. Another study done by Aspenberg et al. also showed a positive result, with a clinically approved dose of 20 µg of teriparatide significantly reducing the median time required for healing compared with placebo treatment. The study found that a 20 µg dose of teriparatide had a positive effect on healing, while a 40 µg dose did not. Additionally, the 20 µg dose was found to have a highly significant impact on reducing the median time to healing compared to a placebo [12]. Bhandari et al. also conducted a prospective study which yielded a positive result [14].

A prospective study done by Kim et al. 2018, showed a negative result. The study found no difference between the two groups in terms of radiographic fracture healing at the final follow-up. The conclusion of the study was that based on the patients studied, short-term use of teriparatide did not reduce pain, improve radiographic signs of fracture healing, or decrease the rate of postoperative complications compared to a placebo in patients with intertrochanteric fractures [24].

A prospective study done by Saraf et al. showed a positive result. The study found that for delayed union fractures, the use of teriparatide was associated with a shorter healing time compared to the group treated with a placebo [36]. Another prospective study done by Kastirr et al. also showed a positive result after an average of 4.1 ± 1.5 (two to six) months after PTH treatment. The study found that 30 out of 32 patients who were treated with teriparatide for delayed union fractures experienced a stable bone consolidation at the nonunion site and were able to regain full weight-bearing capacity of the affected limb without experiencing pain. The mean time between the initial fracture and the PTH treatment was 24.3 ± 17.8 months [23].

Retrospective Studies

We included two retrospective studies in this review, both of which showed significant positive results by showing faster healing rates and lesser complication rates. The first retrospective study, done by Huang et al. (2015), showed that the mean union time was longer (14.3 ± 2.8 weeks versus 11.2 ± 1.6 weeks), the sliding of the lag screw was greater (9.6 ± 5.3 mm versus 2.2 ± 1.4 mm,  p<0.001), there was more femoral shortening, and the study found that varus collapse was more severe in hips that did not receive teriparatide treatment compared to hips that were treated with teriparatide. The main conclusion of the study is that teriparatide improves the healing of fractures, reduces surgical and healing complications, and leads to better clinical outcomes at three and six months after surgery in elderly patients with unstable pertrochanteric fractures.

The second retrospective study, done by Kim (2019), showed that the mean time to fracture healing postoperatively was 14.8 weeks (SD 7.1) and 12.1 weeks (SD 6.4) in the placebo group and teriparatide group, respectively. The frequency of patients reporting postoperative complications was also markedly reduced in the teriparatide-treated groups. The overall conclusion is that short-term daily teriparatide used for osteoporosis treatment improved radiographic fracture healing of a hip fracture and reduced complication rates.

Case Reports and Series

According to Johansson's study, there were no improvements in radiographic signs of healing or clinical improvement in the group treated with short-term (four weeks) daily teriparatide (20 μg/day) in proximal humerus fractures. However, Baillieul et al. reported that when a three-month-old delayed union was started on daily treatment by teriparatide (20 micrograms per day, subcutaneous injection), after six months of treatment, the patient was asymptomatic and on CT, signs of bone re-mineralization were described [13]. Ochi et al. also reported a case of a 74-year-old woman with nonunion of a periprosthetic fracture after total knee arthroplasty, in whom bone union could not be achieved even after she underwent internal fixation and bone grafting twice; however, successful bone fusion was achieved after simple once-weekly administration of teriparatide for six months [31]. In a case series described by Saraf et al. done on 20 patients with fractures, all patients in the study were given 20 µg of teriparatide injections daily. The duration of treatment varied between two to four months, depending on the type of fracture and the time required for radiographic evidence of union. The study found that fractures treated with 20 µg of teriparatide showed early signs of union with significant callus formation, a decrease in the time required for radiographic union, and early rehabilitation of patients in all three groups [36].

Tsa and Hu reported a case of a 60-year-old woman with a right femoral shaft fracture who immediately underwent closed reduction and internal fixation surgery with intramedullary nailing showed no signs of healing for six months, and her condition was diagnosed as atrophic nonunion. Subsequently, teriparatide 20 micrograms/day was administered for six months subcutaneously, and a complete union was observed at the fracture site six months after discontinuing teriparatide [39]. Kastirr et al. also reported on a patient with a fracture in the lower leg that had not yet consolidated after seven months. After receiving therapy with 20 µg of teriparatide per day for eight weeks, the fracture had consolidated, and the patient was able to regain full weight-bearing capacity of the leg without experiencing pain and without any reported side effects. These cases suggest that teriparatide may be effective in promoting the healing of fractures that have not yet consolidated, and further research is needed to establish the efficacy and safety of teriparatide in these cases [22].

Matsumoto described a case report of the successful use of teriparatide to treat delayed union of a spine fracture in a patient with DISH without surgical intervention. The patient, a 70-year-old man, was treated with teriparatide and achieved union in two months without experiencing any adverse events. Six months after starting teriparatide, additional bone formation was observed, and the patient's lumbar instability had resolved [28]. Fukuda et al. also reported a 74-year-old female patient with atypical femoral fracture delayed unions, showing a union of bilateral femurs after three months of weekly once administration of teriparatide [17]. Chintamaneni S reported a case of the first successful use of teriparatide in the healing of a sternal nonunion fracture [16]. Many other case reports and case series have described the successful use of teriparatide in improving callus formation and healing fracture delayed union and nonunions, with no reported side effects [30-41]. These studies suggest that teriparatide may be effective in promoting the healing of fractures that have not yet consolidated, and further research is needed to establish the efficacy and safety of teriparatide in these cases.

Limitations

Our systematic review has limitations as we only used articles in English. This study contains case studies and case series as a majority, and only five randomized controlled trials were included. More prospective studies are needed to strengthen the evidence of teriparatide's efficacy in delayed treatment and nonunion.

## Conclusions

By examining the various outcomes of teriparatide use, the current review intends to evaluate the effectiveness and safety of teriparatide in delayed union and nonunion, and its overall impact on fracture healing and callus formation. This systematic review has taken into consideration case studies, case series, and retrospective and prospective studies.

Existing evidence demonstrates that teriparatide may help in improving callus formation and promote fracture healing in cases of delayed union and nonunion. The use of this drug is highly suggested, as it is safe and highly effective when compared to surgical approaches risking postoperative infections and many other complications. Many more prospective studies are needed to strengthen the evidence regarding the efficacy of teriparatide in this regard.
